# Unresolving Pneumonia in Type 2 Diabetes: Case Report of Polymicrobial Lung Disease

**DOI:** 10.1002/ccr3.72720

**Published:** 2026-05-14

**Authors:** Micheal Collins Segawa

**Affiliations:** ^1^ College of Health Sciences Makerere University Kampala Uganda

**Keywords:** *Candida glabrata*, *Klebsiella pneumoniae*, multidrug‐resistant infection, polymicrobial lung infection, type 2 diabetes mellitus, unresolving pneumonia

## Abstract

Polymicrobial pneumonia caused by the simultaneous infection with multiple pathogens is increasingly recognized in individuals with comorbidities such as type 2 diabetes mellitus (T2DM). The immunocompromised state associated with T2DM predisposes patients to both typical and opportunistic infections, including multidrug‐resistant bacteria and fungi. Prompt diagnosis and organism‐specific treatment are critical for optimal outcomes. We report the case of a 53‐year‐old male with newly diagnosed T2DM who presented with a 3‐month history of left‐sided chest pain, dry cough, dyspnoea, and persistent fever. Initial empirical antibiotic therapy was ineffective. Imaging revealed a massive loculated left pleural effusion with compressive atelectasis. Pleural fluid analysis identified a polymicrobial infection involving multidrug‐resistant *Klebsiella pneumoniae* , *Enterobacter* spp. with inducible AmpC resistance, and azole‐resistant *Candida glabrata*. Intravenous meropenem, echinocandin antifungal therapy, and subsequent oral fluconazole were recommended for successful treatment. Glycaemic control was concurrently optimized with insulin therapy. This case emphasizes the importance of considering polymicrobial infections in diabetic patients presenting with unresolving pneumonia. Early use of imaging and pleural fluid analysis enabled prompt identification of the causative organisms, guiding tailored antimicrobial therapy. Multidisciplinary management is essential in improving outcomes in such complex cases.

## Key Clinical Message

Polymicrobial infection, including opportunistic organisms, should be considered in diabetic patients with nonresolving pneumonia. Early targeted therapy based on microbiological findings is crucial for improved outcomes.

## 1 Introduction

Polymicrobial pneumonia is defined as pneumonia due to more than one pathogen, such as bacterial, fungal, and viral organisms. It is increasingly recognized as a cause of severe pneumonia, especially in individuals with underlying comorbidities such as diabetes mellitus [1, 2, 3]. Diabetes mellitus type 2 (T2DM) is associated with impaired immune function, including decreased neutrophil chemotaxis and phagocytosis, placing patients at a higher risk of both typical and opportunistic infections [4].

*Klebsiella pneumoniae* and other Enterobacteriaceae are frequently implicated in hospital‐ and community‐acquired pneumonia in diabetic patients and can cause rapidly progressing disease due to their virulence and antibiotic resistance mechanisms [5]. Similarly, *Candida glabrata is an emerging pathogenic yeast commonly found in patients with diabetes mellitus. C* *. glabrata* infections are difficult to treat and are often resistant to many azole antifungal agents, especially fluconazole [6, 7].

This case report highlights a rare presentation of polymicrobial pneumonia in a patient with newly diagnosed T2DM involving *Klebsiella pneumoniae* , *Enterobacter* spp., and *Candida glabrata*. The complexity of diagnosis and management in such settings highlights the importance of timely imaging, microbiological evaluation, and tailored antimicrobial therapy.

## 2 Case Report

### 2.1 Case History

A 53‐year‐old male newly diagnosed with type 2 diabetes mellitus (T2DM) was referred from a regional referral hospital for tertiary care with a provisional diagnosis of suspected pulmonary malignancy. He had a three‐month history of left‐sided chest pain and a dry cough, without haemoptysis. He was unresponsive to empirical ceftriaxone, administered before referral. His condition progressively worsened with persistent fevers and dyspnoea. He was a social drinker with no history of smoking.

### 2.2 Examination

On examination, he appeared ill and was receiving oxygen at 5 L/min. He was febrile (38.1 C), tachycardic (114 bpm), normotensive (BP 132/87 mmHg), and had a respiratory rate of 24 bpm. There were reduced breath sounds and stony dull percussion over the left lung fields. No oral thrush, jaundice, lymphadenopathy, or peripheral oedema were present. Other systemic examinations were unremarkable.

### 2.3 Differential Diagnosis

The patient's presentation with chronic left‐sided chest pain, dry cough, persistent fever, and progressive respiratory symptoms raised several possible differential diagnoses. Pulmonary tuberculosis was a major consideration due to chronic dry cough, chest pain, fever, and poor response to antibiotics like ceftriaxone. Empyema thoracis or a complicated parapneumonic effusion was suggested due to fever, dyspnoea, reduced breath sounds, and stony dullness. Pulmonary fungal infections were considered, particularly in the context of newly diagnosed diabetes mellitus, which may predispose individuals to opportunistic infections, and symptoms worsening despite antibiotic therapy. Lung malignancy Justification, given Age > 50, chronic symptoms, chest pain, dry cough, and unresolving symptoms despite antibiotics. Dullness to percussion and decreased breath sounds could suggest a malignant pleural effusion. Nonsmoker status makes this less likely, but still possible (especially adenocarcinoma).

### 2.4 Investigations

**FIGURE 1:**
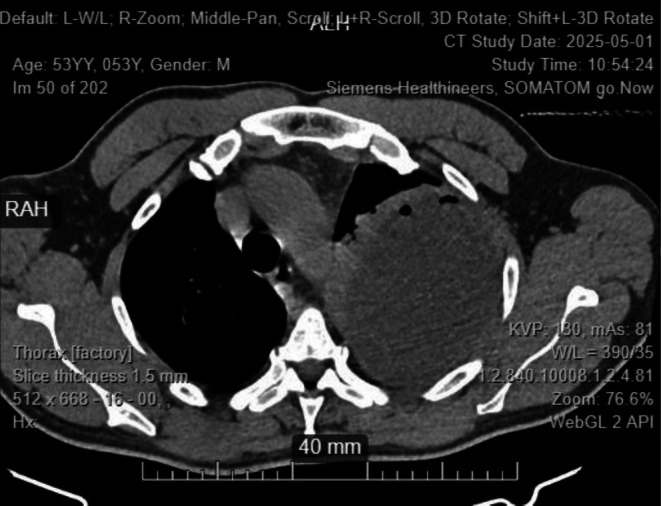
CT image.

**FIGURE 2:**
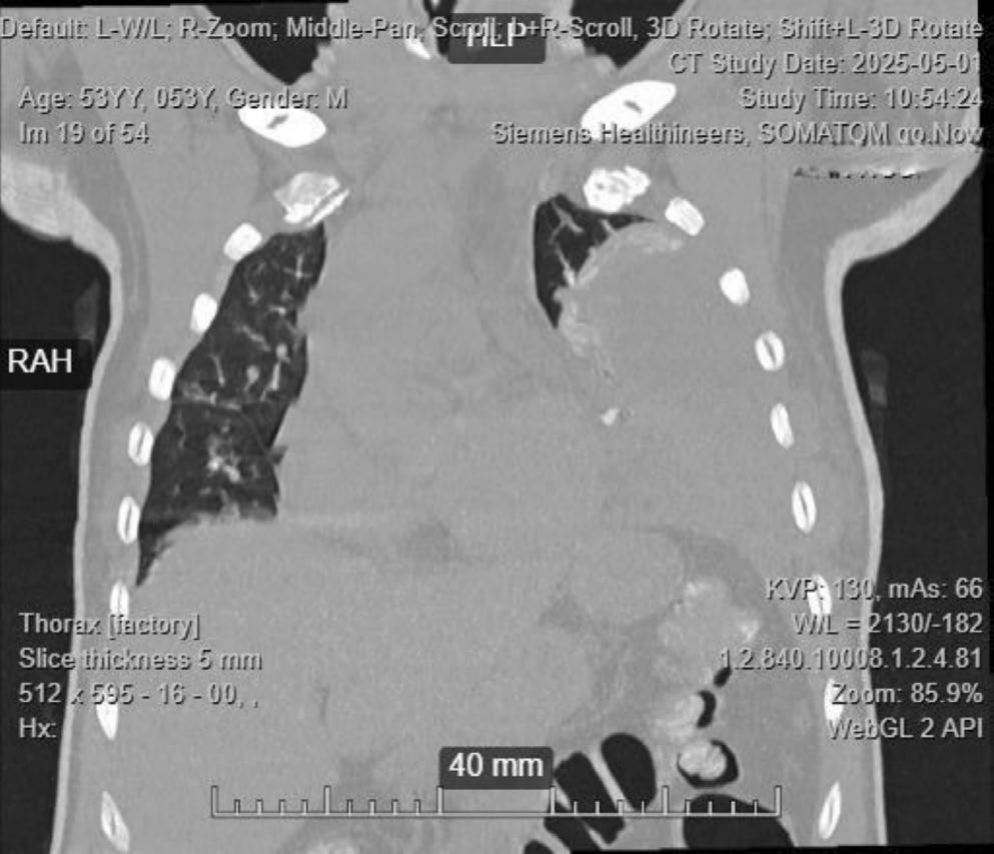
CT images.

**FIGURE 3:**
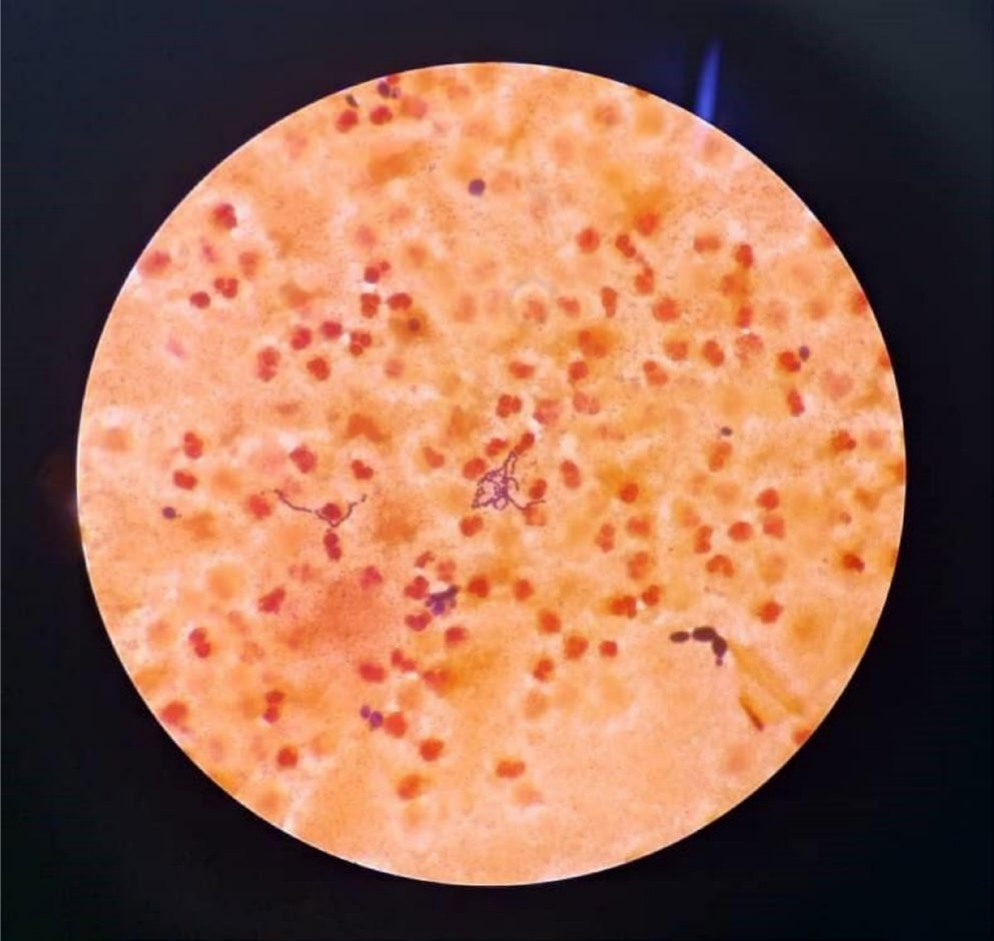
Direct microscopy of gram‐staining.

**FIGURE 4:**
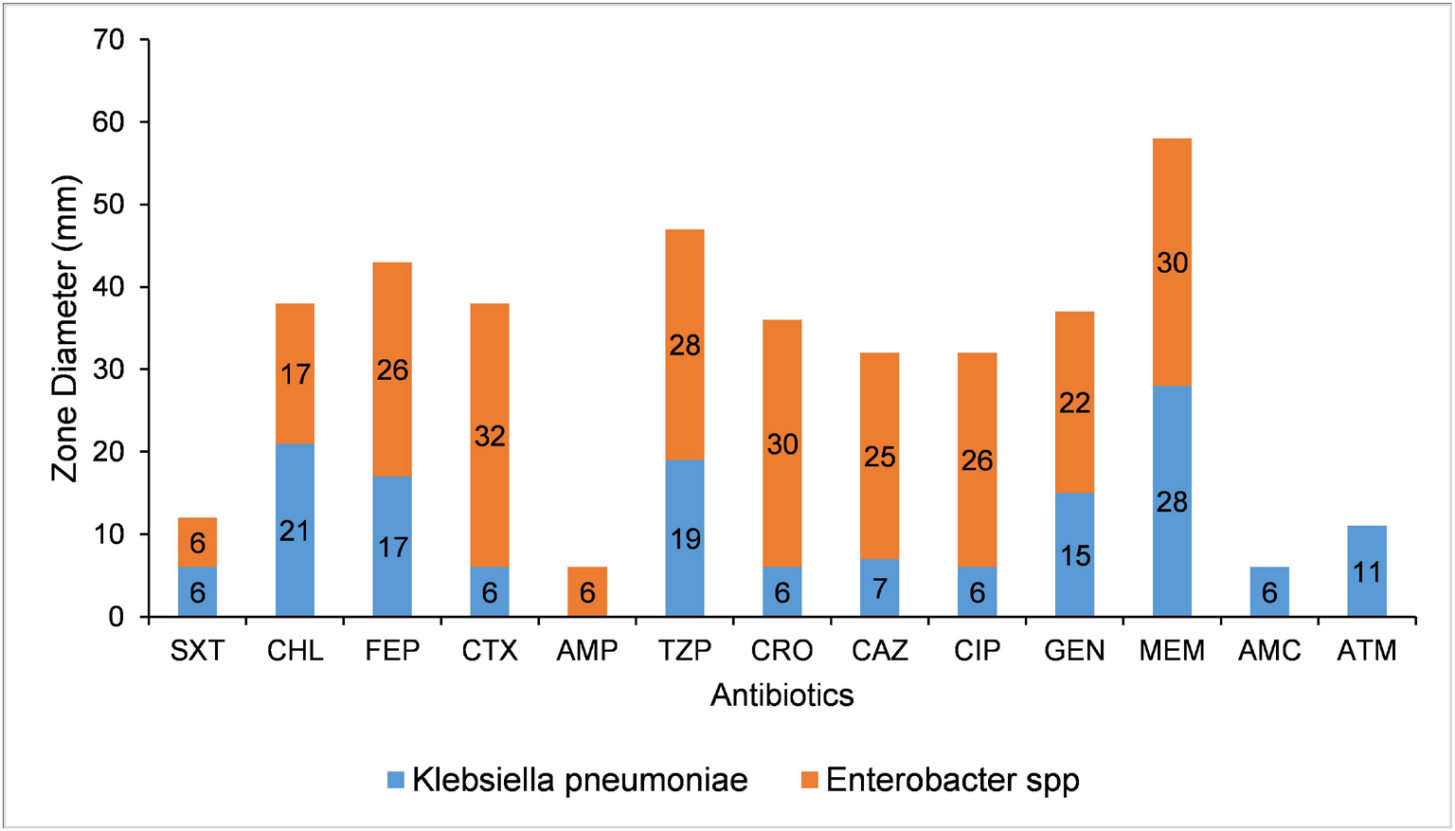
Antibiotic disc diffusion zone diameters for bacterial isolates from pleural fluid. AMC, amoxicillin‐clavulanate (Augmentin); AMP, ampicillin; ATM, aztreonam; CAZ, ceftazidime; CHL, chloramphenicol; CIP, ciprofloxacin; CRO, ceftriaxone; CTX, cefotaxime; FEP, cefepime; GEN, gentamicin; MEM, meropenem; SXT, sulfamethoxazole‐Trimethoprim; TZP, piperacillin‐tazobactam.

**FIGURE 5:**
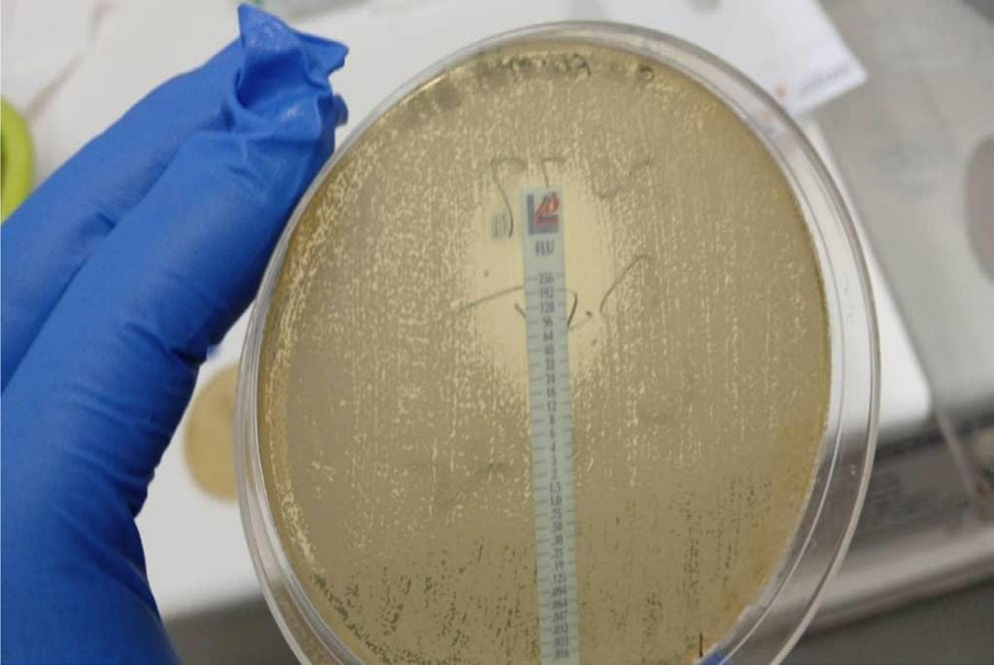
Antifungal susceptibility testing of *Candida glabrata* using gradient strips.

Random blood sugar was 16.7 mmol/L, and TB‐gene Xpert results were negative. A contrasted chest CT revealed massive loculated left pleural effusion with compressive atelectasis of the left lung lobes, small right pleural effusion, patchy consolidations in the right lower and middle lobes, but no evidence of intrathoracic mass (Figures 1 and 2). Pleural fluid aspirate analysis, culture, and sensitivity revealed 4+ inflammatory cells, 3+ budding yeast cells, 3+ gram‐positive cocci in chains, and 2+ gram‐negative rods on Gram‐staining (Figure 3), no acid‐fast bacilli on ZN stain, and no malignancy on cytological analysis. Culture and sensitivity results confirmed *Klebsiella pneumoniae* multidrug‐resistant (MDR), resistant to cephalosporins, aminoglycosides, and fluoroquinolones, sensitive to meropenem. *Enterobacter* spp. with inducible AmpC resistance, sensitive to piperacillin‐tazobactam and meropenem (Figure 4). *Candida glabrata* resistant to amphotericin B, susceptible, dose‐dependent to fluconazole (Figure 5).

### 2.5 Treatment

Following the CT‐scan results, the patient was started on empirical IV Piperacillin and tazobactam injection and oral Azithromycin before thoracocentesis and pleural fluid analysis. Insulin was given to return the blood sugar to normal. After the pleural fluid analysis, the recommended treatment for the patient was intravenous meropenem (extended infusion every 8 h), intravenous echinocandin for 14 days, then transitioned to high‐dose oral fluconazole for 4 weeks. The patient showed significant clinical improvement following initiation of targeted antimicrobial therapy, with resolution of fever and dyspnoea. The patient was successfully discharged on oral fluconazole therapy with outpatient follow‐up.

## 3 Discussion

This case illustrates the diagnostic and therapeutic challenges of managing polymicrobial pneumonia in the setting of newly diagnosed T2DM. The patient presented with nonresolving pneumonia despite broad‐spectrum antibiotics, raising suspicion for atypical pathogens or mixed infections, both of which are more likely in individuals with impaired immunity [4]. Polymicrobial pneumonia is often underdiagnosed due to the limitations of routine culture techniques and the assumption that a single pathogen is responsible for the clinical picture [3]. In this case, the combination of gram‐negative bacteria and yeast in pleural fluid highlights the need for a high index of suspicion in at‐risk populations. Diabetes alters the respiratory tract microbiome, promoting colonization and infection with resistant pathogens such as *Klebsiella pneumoniae* and *Enterobacter* spp. [5, 8] Moreover, hyperglycaemia impairs host immune responses and promotes fungal growth, thereby facilitating the pathogenicity of *Candida glabrata*, which is increasingly recognized for its multidrug resistance and ability to cause invasive infections [9, 10].

Imaging, particularly CT scanning, was crucial in identifying a massive loculated pleural effusion and consolidations suggestive of empyema. This guided further diagnostic steps, including thoracentesis and pleural fluid analysis. Gram staining and cultures helped identify the polymicrobial etiology and informed the escalation to appropriate antimicrobial therapy. The identification of extended‐spectrum beta‐lactamase (ESBL)‐producing *Klebsiella* and AmpC‐producing *Enterobacter* spp. necessitated the use of carbapenems, in line with the Infectious Diseases Society of America (IDSA) guidelines [11].

Treatment of *Candida glabrata* is particularly challenging due to its reduced susceptibility to fluconazole and inherent resistance to amphotericin B. Echinocandins are the first‐line agents in such scenarios, especially in immunocompromised hosts [10, 12]. In this case, intravenous echinocandin followed by high‐dose fluconazole was used, adhering to standard antifungal stewardship principles.

This report underscores the importance of considering polymicrobial infections in immunocompromised patients presenting with unresolving pneumonia and highlights the need for multidisciplinary approaches involving imaging, microbiology, and individualized antimicrobial regimens.

## 4 Conclusion

These findings suggest a polymicrobial infection in a patient with immunosuppression related to type 2 diabetes. In a diabetic, *Candida glabrata* is more likely to be clinically significant rather than a contaminant.

## Author Contributions

**Micheal Collins Segawa:** conceptualization, data curation, software, validation, visualization, writing – original draft, writing – review and editing.

## Funding

The author has nothing to report.

## Disclosure

The author read and approved the final version of the manuscript.

## Ethics Statement

The author has nothing to report.

## Consent

Written informed consent was obtained from the patient before reporting this case.

## Data Availability

The experimental data used to support the findings of this study are available from the corresponding author upon reasonable request.

## References

[1] C. Cillóniz , S. Ewig , M. Ferrer , et al., “Community‐Acquired Polymicrobial Pneumonia in the Intensive Care Unit: Aetiology and Prognosis,” Critical Care 15, no. 5 (2011): R209, 10.1186/cc10444. 21914220 PMC3334753

[2] L. M. Filkins and G. A. O'Toole , “Cystic Fibrosis Lung Infections: Polymicrobial, Complex, and Hard to Treat,” PLoS Pathogens 11, no. 12 (2015): e1005258, 10.1371/journal.ppat.1005258. 26719892 PMC4700991

[3] S. Jain , W. H. Self , R. G. Wunderink , et al., “Community‐Acquired Pneumonia Requiring Hospitalization Among U.S. Adults,” New England Journal of Medicine 373, no. 5 (2015): 415–427, 10.1056/NEJMoa1500245. 26172429 PMC4728150

[4] L. M. A. J. Muller , K. J. Gorter , E. Hak , et al., “Increased Risk of Common Infections in Patients With Type 1 and Type 2 Diabetes Mellitus,” Clinical Infectious Diseases 41, no. 3 (2005): 281–288, 10.1086/431587. 16007521

[5] R. Podschun and U. Ullmann , “Klebsiella spp. as Nosocomial Pathogens: Epidemiology, Taxonomy, Typing Methods, and Pathogenicity Factors,” Clinical Microbiology Reviews 11, no. 4 (1998): 589–603, 10.1128/CMR.11.4.589. 9767057 PMC88898

[6] M. G. Frías‐De‐león , R. Hernández‐Castro , E. Conde‐Cuevas , et al., “ *Candida glabrata* Antifungal Resistance and Virulence Factors, a Perfect Pathogenic Combination,” Pharmaceutics 13, no. 10 (2021): 1529, 10.3390/pharmaceutics13101529. 34683822 PMC8538829

[7] P. L. Fidel , J. A. Vazquez , and J. D. Sobel , “Candida Glabrata: Review of Epidemiology, Pathogenesis, and Clinical Disease With Comparison to *C. albicans* ,” Clinical Microbiology Reviews 12, no. 1 (1999): 80–96, 10.1128/cmr.12.1.80. 9880475 PMC88907

[8] F. Fiocca Vernengo , I. Röwekamp , L. Boillot , et al., “Diabetes Impairs IFNγ‐Dependent Antibacterial Defense in the Lungs,” Mucosal Immunology 18, no. 2 (2025): 431–440, 10.1016/j.mucimm.2024.12.015. 39746547

[9] M. A. Pfaller and D. J. Diekema , “Epidemiology of Invasive Candidiasis: A Persistent Public Health Problem,” Clinical Microbiology Reviews 20, no. 1 (2007): 133–163, 10.1128/CMR.00029-06. 17223626 PMC1797637

[10] P. G. Pappas , C. A. Kauffman , D. R. Andes , et al., “Clinical Practice Guideline for the Management of Candidiasis: 2016 Update by the Infectious Diseases Society of America,” Clinical Infectious Diseases 62, no. 4 (2015): e1–e50, 10.1093/cid/civ933. 26679628 PMC4725385

[11] P. D. Tamma and J. Rodriguez‐Baňo , “The Use of Noncarbapenem β‐Lactams for the Treatment of Extended‐Spectrum β‐Lactamase Infections,” Clinical Infectious Diseases 64, no. 7 (2017): 972–980, 10.1093/cid/cix034. 28362938 PMC5848369

[12] M. Hoenigl , J. Salmanton‐García , T. J. Walsh , et al., “Global Guideline for the Diagnosis and Management of Rare Mould Infections: An Initiative of the European Confederation of Medical Mycology in Cooperation With the International Society for Human and Animal Mycology and the American Society for Microbiology,” Lancet Infectious Diseases 21, no. 8 (2021): e246–e257, 10.1016/S1473-3099(20)30784-2. 33606997

